# Pulmonary Salivary Gland Tumor, Mucoepidermoid Carcinoma: A Literature Review

**DOI:** 10.1155/2022/9742091

**Published:** 2022-11-02

**Authors:** Shumin Hu, Jiali Gong, Xiu Zhu, Hongyang Lu

**Affiliations:** ^1^The Second Clinical Medical College, Zhejiang Chinese Medical University, Hangzhou 310053, China; ^2^Zhejiang Key Laboratory of Diagnosis & Treatment Technology on Thoracic Oncology (Lung and Esophagus), Cancer Hospital of the University of Chinese Academy of Sciences (Zhejiang Cancer Hospital), Hangzhou 310022, China; ^3^Department of Thoracic Medical Oncology, Cancer Hospital of the University of Chinese Academy of Sciences (Zhejiang Cancer Hospital), Zhejiang 310022, China; ^4^Institute of Basic Medicine and Cancer (IBMC), Chinese Academy of Sciences, Beijing 310022, China; ^5^Department of Pathology, Cancer Hospital of the University of Chinese Academy of Sciences (Zhejiang Cancer Hospital), Zhejiang 310022, China

## Abstract

Pulmonary mucoepidermoid carcinoma (PMEC) is the most common malignant salivary gland tumor in the lungs and accounts for 0.1-0.2% of all lung malignancies in adults. It has no specific epidemiological or clinical characteristics. Correct diagnosis requires the combined examinations of images, laboratories, pathology, and immunohistochemistry (IHC) as well as molecular characteristics. PMEC tumors are characterized by squamous, intermediate, and mucus-secreting cells. Currently, histological appearance, mitotic frequency, cellular atypia, and necrocytosis allow the classification of PMEC into low grade or high grade. Molecular changes are crucial to pathological diagnosis. The driver of PMEC seems to be the fusion protein MECT1-MAML2 that is generated from a genetic mutation in *t* (11; 19) (*q*21; *p*13), while other gene mutations are also reported. However, no treatment of PMEC exists so far; surgical excision is still the primary treatment, while the efficacies of chemotherapy or radiotherapy are undefined. Tyrosine kinase inhibitor (TKI) therapy and immunotherapy showed to have significant therapeutic effects but require more investigation and better understanding. This review focuses on the clinical characteristics, imaging and pathologic features, immunohistochemical examination, mutation analysis, differential diagnosis, prognosis, and treatment of PMEC.

## 1. Introduction

In 1952, Smetana et al. [1] first reported pulmonary mucoepidermoid carcinoma (PMEC) as a scarce malignant neoplasm in the lungs that accounted for 0.1–0.2% of all lung malignancies in adults [2–6]. PMEC, the most common primary salivary gland carcinoma (SGC) in the lung, originates from the minor salivary glands in the submucosa of large airways [7]. PMEC occurs in any age group; some studies reported its occurrence primarily in younger adults under 50 [8]. The clinical symptoms and epidemiological characteristics of PMEC are not specific and representative. Accordingly, correct diagnosis of PMEC requires the combination of clinical characteristics, histopathological examination, immunohistochemistry (IHC), and molecular mutational analysis. Tumors caused by PMEC are characterized by the histopathology of squamous epithelial, mucous, and intermediate cells. The classification of PMEC into high grade or low grade depends on histological appearances, mitotic frequencies, cellular atypia, and necrocytosis [2, 8, 9].

The most common genetic change is *t* (11; 19) (*q*21; *p*13), which generates the fusion protein MECT1-MAML2. Since MECT1-MAML2 fusion was demonstrated to be present in more than 66% of all PMEC cases [8, 10, 11], it was proposed to drive PMEC progress [7]. Several other gene mutations were also reported. For example, a mutation in the gene for the epidermal growth factor receptor (EGFR) was observed in patients with PMEC [12]. These genomic alterations are potential for selecting therapy.

Up to now, there is still no consolidated strategy for the therapy of PMEC, and the complete surgical resection is recognized as the primary therapeutic method. The effects of chemotherapy or radiotherapy as valid therapies have not been shown yet [13]. Several case reports show that EGFR-inhibiting agents (gefitinib and erlotinib) have efficacies in patients with PMEC [14–16]. An immunotherapy approach for PMEC is limited so far and needs to be explored in-depth; therefore, advanced research currently prioritizes targeted therapy and immunotherapy.

## 2. Clinical Characteristics

From an epidemiological perspective, PMEC is a rare pulmonary tumor that appears in a wide range of ages. Generally, the age of onset ranges from 7 to 87 years, and the mean age is approximately 50–60 years. The incidence of PMEC in patients over 75 years is rare, and Abdalla et al. [17] reported a rare case of an 81-year-old male with PMEC. In terms of gender, although some studies suggested that the incidence in males is higher than in females [2, 18, 19], most reports demonstrated that the incidence between males and females possesses a similar distribution [8, 20, 21]. Interestingly, only a few patients stated they were smoking. Hence, there does not seem to be a correlation between PMEC onset and smoking, and this needs to be confirmed in advanced studies.

PMEC is not accompanied by any specific clinical symptom, while the most common symptom is cough. Other symptoms include blood-tinged or whitish sputum, fever, hemoptysis, chest tightness, chest pain, hoarseness, and dyspnea; yet some patients have no obvious symptoms and are only diagnosed during a physical examination. Three patients were even diagnosed with PMEC upon hospital admission because of a cough [17, 22, 23]. Interestingly, the clinical symptom of a PMEC patient, as well as its frequency and extent, depends on the position of the lesion. Tumors located in the central bronchus will appear as obstructive airway symptoms and primarily manifest as cough, dyspnea, or asthma. On the other hand, 85% of PMEC appears in the peripheral lung. These tumors may manifest as cough, chest pain, and pulmonitis [24, 25] or can be asymptomatic and only be found in physical examinations. These results suggest that PMEC has no obvious symptoms, while it can be easily misdiagnosed or overlooked. Hence, due to the challenge of correctly identifying this benign disease, it is necessary to raise awareness of PMEC to decrease the rate of misdiagnosis.

## 3. Imaging Examination

Computed tomography (CT) is a vital and necessary approach for the diagnosis and differential diagnosis of PMEC. CT is a noninvasive and convenient technique that can be adopted to explore suspected trachea and lung lesions. Most studies based on CT describe PMEC as a well-defined mass characterized as the central or hilar type, oval or round shape, with smooth margins and marked enhancement [26–28]. Wang et al. [29] used CT images to distinguish between low-grade and high-grade PMEC. Low-grade PMEC usually manifests as a central bronchial mass with marked homogeneous enhancement. In contrast, high-grade PMEC tends to be in the periphery and manifests as a lobular and heterogeneous mass with poorly defined margins and minor enhancement. Additionally, Cheng et al. [27] used multisection-computed tomography (MSCT) to reveal an oval or lobulated, mildly enhanced mass with calcification and mucus lakes that may indicate PMEC. Similarly, Park et al. [30] applied ^18^F-FDG PET/CT to predict the pathologic grade and prognosis of PMEC. The authors concluded that patients with SUV_max_ greater than 6.5 tend to have high-grade PMECs, lymph node metastasis, and recurrences. The size of tumors varies approximately from 1 cm to 10 cm. The majority of literature reports indicate tumor sizes of 2.0–3.0 cm.

The preferential location of PMEC distribution is rather varied as shown by several studies. For example, Zhang et al. [31] and Salem et al. [10] showed that tumors often occur in the left lower lobe and right upper lobe. Qiu et al. [20] also observed that PMEC commonly occurs in the upper lobe and lower lobe rather than in the bronchus. However, Cheng et al. examined 43 patients with PMEC and observed that PMECs more likely occur in the right lower lobe and left upper lobe [27]. In addition, Huo et al. [8] investigated 26 patients with PMEC and found that tumors were rather located in the segmental bronchus and lobe. Hence, it can be concluded that PMEC tumors can occur in any lobe of the lung with no preference to its location.

## 4. Pathology and Immunohistochemistry Examinations

Grossly, PMEC tumors are tan or light brown polypoid mass. The central bronchus may be present as an exophytic tumor and nearly completely occlude the bronchial lumen [32]. PMEC are histopathologically defined by a combination of squamous epithelial, mucous, and intermediate cells with defects in keratinization. The standard classifies PMEC into low-grade and high-grade tumors, depending on histological appearance, mitotic frequency, cellular atypia, and necrocytosis [2, 8, 9]. Low-grade PMECs are combinations of all three cell types without any specific differentiation, comprised of cystic changes prevailingly. Mitotic figures, nuclear atypia, and necrosis are rarely observed. Microscopic invasion into pulmonary parenchyma is unusual [33]. High-grade PMEC primarily consists of squamous epithelial and intermediate cells with a small number of mucous cells, with a presentation of predominantly solid pattern growth, continual necrosis, mitoses (more than 4/10 high-power fields), or distinct atypia [8, 10, 27, 29, 34]. Chin et al. [18] observed that high-grade tumors had a higher proportion of squamous epithelium. Besides, invasion into the adjacent pulmonary parenchyma and regional lymph node involvement are more frequent in high-grade PMEC [9, 21, 33, 35]. In the majority of the series, the authors concluded that the pathological classification of PMEC is significant for diagnosis, treatment, and prognosis. Patients with low-grade PMEC have a better survival outcome compared with those with high-grade PMEC [8, 9, 13, 19, 25, 29, 36, 37]. In addition, Wang et al. [29] compared the association between pathologic grade and predilection sites of PMEC and observed that low-grade PMEC usually appears in the central lung, whereas high-grade PMEC often occurs in the peripheral lung.

The IHC characteristics of PMEC are retrospectively analyzed and summarized in Table 1. Here, the positive percentage of p63, CK7, Muc5Ac, p40, and CK5/6 was found to be 58/58 (100%), 33/33 (100%), 26/26 (100%), 52/54 (96.3%), and 3/6 (50%), respectively. Napsin A, TTF-1, and human epidermal growth factor receptor 2 (HER2) were all negative. Most of the studies reported that p63 and p40 are expressed, while TTF-1 and Napsin A are negative in PMEC. However, Zhang et al. [31] reported that some cases were positive for TTF-1 and napsin A, which is inconsistent with the results of the majority of reports. In low-grade cases, the Ki-67 labeling index was less than 10%, while in cases of high-grade PMEC, the index was more than 20% [8, 19, 23]. Hence, the Ki-67 labeling index potentially be used as an auxiliary index for differentiating high-grade from low-grade PMEC.

P63 commonly supports the diagnosis of squamous cells. Therefore, p63 is confirmed to be positive in PMEC. It is yet generally positive in adenosquamous carcinoma and squamous cell carcinoma, so it may lead to misdiagnosis [8, 38]. P63 could be adopted to distinguish PMEC from other salivary gland tumors, especially acinic cell carcinoma, since p63 is generally negative in acinic cell carcinoma [39]. P40 is another IHC marker adopted to diagnose PMEC. Roden et al. [11] observed that the expression pattern between p40 and p63 was semblable in most cases, while the p40 expression score was lower than p63 in nearly one-quarter of PMEC. A few p40-negative cases have focal p63 expression. Consequently, they realized p63 could be a more sensitive marker. Despite, it has recently been proposed to be more specific than p63 for squamous differentiation [38]. Since TTF-1 is always negative in PMEC, it is conducive to distinguishing PMEC from primary pulmonary adenosquamous carcinoma and adenocarcinoma [8, 11, 13].

*HER2* gene and protein change are molecular basics for target therapy in cancer. As a whole, it is reported HER2 gene amplification in 1.0%–14.3% by fluorescence in situ hybridization (FISH) and protein overexpression in 4.3%–38% by IHC of salivary mucoepidermoid carcinoma (MEC) [40–43]. In addition, both *HER2* amplification and protein overexpression were also reported to be associated with high-grade tumors [40, 42]. One patient with metastatic MEC expressed HER2 positive achieved therapeutic response to trastuzumab [44]. Therefore, Clauditz et al. [40] suggested that IHC and FISH analyses of HER2 should be applied in the cases of recrudescent and/or metastatic disease. Until now, only a few studies have investigated the expression of HER2 in PMEC, and in Table 1, HER2 is negative in all cases (0/26). However, the detection and analysis of HER2 should be explored in larger samples of PMEC.

In summary, the combined detection of p63, p40, CK5/6, CK7, Ki-67 labeling index, the absence of TTF-1, and Napsin A may be an auxiliary diagnostic index of PMEC. HER2 detection (protein overexpression and gene amplification) could be a necessary complement.

## 5. Molecular Characteristics

The mutation *t* (11; 19) (*q*21; *p*13) generating the MECT1-MAML2 fusion protein has been demonstrated to be the specific genetic event for PMEC onset [45]. The rearrangement is fused by mucoepidermoid carcinoma translocated 1 (MECT1) at 19p13 and mastermind-like 2 (MAML2) at 11q21 [46]. Tonon et al. [47] observed that MECT1-MAML2 activated HES1 transcription to disrupt Notch signaling. Wu et al. [48] also found that this fusion protein activates CREB and thus mimics the constitutive activation of cAMP signaling. Therefore, presence of the MECT1-MAML2 rearrangement can support PMEC diagnosis as this genetic change is found in 66% to 100% of PMEC. Simultaneously, some studies proposed that the MECT1-MAML2 rearrangement is more common in low-grade than in high-grade PMEC. For example, Salem et al. [10] showed that 88% (8/9) of PMEC contained the MECT1-MAML2 fusion protein, of which all had a low-grade morphologic tumor. A study by Huo et al. [8] showed similar results; 83.3% (10/12) of low-grade PMEC contained the MAML2 rearrangement, while only 33.3% (2/6) of high-grade PMEC did. Also, Roden et al. [11] detected the MAML2 rearrangement by FISH and confirmed all 24 cases (3 low, 19 intermediate, and 2 high grade) to be positive.

Little is known about the genomic background of PMEC in addition to MECT1-MAML2 translocations. Wang et al. [49] employed a comprehensive genomic profiling to investigate salivary mucoepidermoid carcinomas (3 high-grade PMECs) and revealed the appearance of diverse genomic alterations. These may bring new targets for an immunological therapy approach. Although the detailed genomic change of PMEC was not reported separately, the authors concluded that the majority of patients had at least one genomic alteration and that the most common genomic alterations were in *CDKN2A* and *TP53*. They also indicated that the frequency of both *PIK3CA* alterations and PI3K pathway activation in high-grade tumors is higher than their frequencies in low-grade tumors. Consequently, more potentially actionable genomic alterations have been observed now that can influence therapy selection.

Overexpression of the EGFR protein was common in most cases of PMEC. On the contrary, amplification or mutation within the tyrosine kinase domain of the EGFR gene has been barely reported [8, 50]. However, Yu et al. [12] unveiled 5 cases (25%) in 20 PMEC patients with an uncommon EGFR mutation (exon 21 L861Q heterozygous mutation). This study proved the appearance of EGFR mutations in PMEC and the L861Q mutation to be the predominant EGFR mutation. Yamamoto et al. [51] reported that in two out of nine (22.2%) patients, EGFR gene abnormalities (exon 21) were detected by the IHC method, and in one (11.1%) patient, the EGFR mutation (exon 21 L858R mutation) was observed by the cycleave method.

## 6. Differential Diagnosis

Distinguishing high-grade PMEC from adenosquamous carcinoma (ASC) is rather challenging due to only minor differences in their IHC and histopathological patterns. A study by Huo et al. [8] misdiagnosed 2 ASCs as PMEC based on the presence of mucous cells, solid nests, and the consistency of IHC-positive results. Only by considering the keratinization and positivity of TTF-1 could the diagnosis be modified. Similarly, Chenevert et al. [52] decided to reclassify their ASC cases due to the presence of dysplastic and/or *in situ* carcinoma in the mucosa and extensive keratinization. Therefore, although the differences between PMEC and ASC are only minor, PMEC rarely shows an expression of keratinization and *in situ* carcinoma and a complete absence of TTF-1.

The incidence rate of primary adenoid cystic carcinoma (PACC) is lower than the rate of PMEC in adults [7]. However, no remarkable difference in the clinical manifestation between PMEC and PACC exists [3]. Comparing the epidemiological characteristics with PACC, patients with PMEC are often of younger age at tumor onset, have smaller tumors, less lymph nodes, or distant metastases, and are more likely to be in the early stage of the disease [3, 53]. There are significant distinctions in the predilection site and features shown in CT. PACC occurs more frequently in the central type (located in the main bronchus or trachea) and appears more often as a lobulated mass. Homogeneous or heterogeneous thickening owing to infiltration of the luminal wall is common. PMEC manifests commonly as the hilar type, concomitant by distant bronchial dilatation with mucoid obstruction. CT findings are more likely to suggest an obstructive airway disease. PMEC is more frequently present as an obvious enhancement than PACC [28, 54]. Kumar et al. [25] observed a similar result; PACC usually occurs in the central airways and main bronchial tube, while PMEC was more frequently located in the lungs. As for the results of the immunohistochemical examination, PACC expresses CD117(c-kit protein) and myoepithelial markers, including pancytokeratin, p63, and CK7. *MYB-NFIB* fusion carcinogens generated by tumor-specific t (6; 9) (q22-23; p23-24) translocation are considered to be specific to PACC [7, 55].

**Figure 1:**
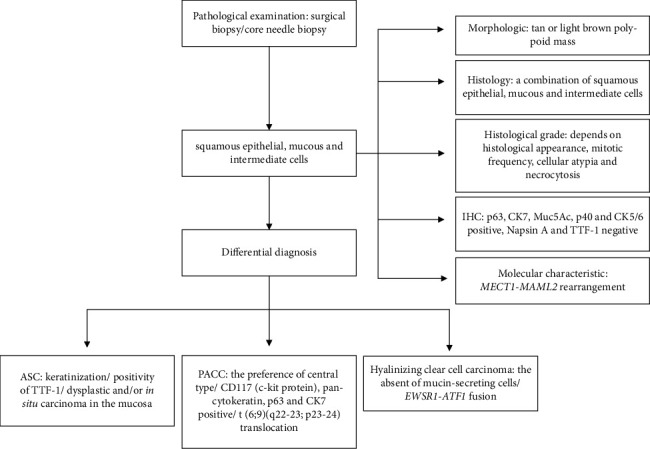
The pathological diagnostic flowchart of PMEC.

The most challenging distinction may be with hyalinizing clear cell carcinoma. Both carcinomas show a similar presence of mucin pools, intracytoplasmic mucin, and hyalinized stroma and immunohistochemically squamous differentiation [56–58]. Takamatsu et al. [57] observed mucin production, yet no mucin-secreting cells were present in hyalinizing clear cell carcinoma. On the contrary, mucin-secreting cells are one of the essential components in PMEC. Furthermore, there is no significant difference in immunohistochemical findings between PMEC and hyalinizing clear cell carcinoma. Similar to PMEC, CK7, CK 5/6, p63, and p40, cytokeratin cocktail is usually positive in hyalinizing clear cell carcinoma, which reveals squamous differentiation. TTF-1, napsin A, CK20, chromogranin, synaptophysin, SMA, HMB45, and melan A, on the other hand, are negative [56, 57]. Ki-67 labeling was ranged from 3 to 10% [57]. Interestingly, molecular analysis can be instrumentally adapted to distinguish PMEC from hyalinizing clear cell carcinoma. *EWSR1-ATF1* fusion is confirmed to be specific in hyalinizing clear cell carcinoma [56–58]. Chapman et al. [58] reported three cases initially diagnosed as PMEC and then demonstrated *EWSR1-CREM* fusion to sustain a diagnosis of hyalinizing clear cell carcinoma. Hence, one can conclude that performing essential cytogenetic and molecular analysis supports a correct differential diagnosis. The pathological diagnostic flowchart of PMEC is shown in Figure 1.

## 7. Treatment

At present, there is no consolidated standard to treat PMEC. However, the principles to treat PMEC are consistent in the domestic and foreign literature. Complete surgical resection is recognized as the predominant therapeutic strategy, which even implies a better survival outcome, especially for stage I–II PMEC [8, 20, 31]. Zhang et al. [31] summarized the median overall survival (OS) of surgery (57/87), radiotherapy (5/87), and others (25/87) as 61 months, 60 months, and 42 months, respectively. It is suggested that the prognosis of surgery is better than that of nonsurgery. Qiu et al. [20] analyzed survival outcomes of treatments in patients with TNM stage I–II and stage III–IV PMEC separately. They concluded that surgical treatments had the highest cancer-specific survival (CSS) compared to other therapies (radiation and/or chemotherapy and surgery plus radiation and/or chemotherapy). Zhang et al. [31] observed that the median OS of surgery (30) and nonsurgery (15) is 60 months and 52 months in stage I-II patients, respectively (*P* = 0.013). Qiu et al. [20] found that local surgical resection should be avoided since patients with local tumor excision had the worst OS and CCS. Besides, concerning PMEC patients with high uptake on PET/CT imaging, Park et al. [30] suggested that mediastinal lymph node dissection and adjuvant therapies were feasible. Consequently, the majority of the studies agree that complete surgical resection and systematic lymph node dissection are essential and necessary for patients who consider surgical treatment.

The efficacy of chemotherapy or radiotherapy remains controversial [13]. However, adjuvant chemotherapy or radiotherapy is probably feasible in patients with high-grade PMEC, especially in cases of extrathoracic invasion. Yan et al. [23] reported a case of comprehensive therapy combining apatinib with fractionated stereotactic radiotherapy. This approach had a therapeutic effect on high-grade PMEC with limited brain metastases, which inspiringly improved brain edema and OS. Sonobe et al. [59] reported a case of a 59-year-old man with high-grade PMEC responding to carboplatin and paclitaxel and suggested that this combination therapy of carboplatin and paclitaxel provided an option for PMEC treatment.

EGFR-tyrosine kinase inhibitor (EGFR-TKI) is the most effective therapy for terminal patients harboring EGFR mutation. Han et al. [15] as well as Rossi et al. [16] reported PMEC patients whose tumor neither showed EGFR protein overexpression nor had EGFR genomic variations by FISH and mutational analysis. However, these patients had a certain response to gefitinib. According to these findings, O'Neill [60] suggested that the tumor-specific *t* (11; 19) (*q*21; *p*13) gene mutation producing the MECT1-MAML2 fusion protein may be an effective target for EGFR-TKI therapy. Lee et al. [14] reported a case of metastatic PMEC with a response to EGFR-TKI erlotinib. It provides a possibility for PMEC treatment. Chen et al. [61] suggested that the MECT1-MAML2 fusion protein upregulates the expression of EGFR ligand amphiregulin (AREG) by binding directly to transcription factor CREB. In the next step, AREG activates EGFR signaling to support the growth and survival of tumor cells, which is why anti-EGFR agents in PMEC-targeted therapy are highly efficient. Additionally, Clauditz et al. [40] observed that the HER2 positive group is characterized by high-level gene amplification; thus, trastuzumab may have a response. According to the immunohistochemical results of Wang et al. [49], PI3K/mTOR inhibitors may have a therapeutic effect, resulting from 52% high-grade tumors were observed gene alternations in PI3K/mTOR pathway. Recently, a multicenter phase 2 study has looked at the effect of nintedanib in patients with recurrent or metastatic salivary gland cancer of the head and neck [62]. This work revealed a promising clinical efficacy and achieved a 75% disease-control rate (15/20) of nintedanib to treat this condition.

The knowledge on immunotherapy for PMEC is limited so far. Undoubtedly, programmed death-1, programmed death ligand-1, and programmed death ligand-2 (PD-1, PD-L1, andPD-L2) are the most studied immune pathway targets in various carcinomas. Some of the literature about salivary MEC could show a directional effect. The expression rates of PD-1, PD-L1, and PD-L2 are summarized in Table 2. In studies with small sample sizes, the positive rate of PD-L1 is approximately 50%–60%, whereas the rate of PD-L2 is rather low. However, contrary results of PD-L1 are reported in large sample studies. Liu et al. [63] found that the intensity of PD-L2 expression had a positive relation with the histological grade. Besides, PD-L2 is likely to be associated with tumor recurrence [63, 64]. It can be predicted that PMEC is a tumor with low expression of PD-L1, whose evasion mechanisms are likely related to PD-L2.

The KEYNOTE-028 phase IB trial measured pembrolizumab against advanced PD-L1-positive SGCs. Among SGC patients (3 MECs), 26 PD-L1 were positively treated with pembrolizumab and the overall response rate (ORR) was 12%. The trial reported that only three patients had partial responses (PRs) [68]. Tumor mutational burden (TMB) can estimate tumor neoantigen load [69]; therefore, cancer with high TMB has strong immunogenicity [70]. The MyPathway trial observed that one high-TMB MEC patient achieved PR with the treatment of atezolizumab [71]. PD-L1 and TMB are reliable biomarkers to evaluate the curative effects of immunotherapy [69], what yet has low-to-moderate immunogenicity in the prevalent MEC. Consequently, its potential as the target for current immunotherapy seems not to bejk remarkable [72], whereas immunotherapy aimed at PD-L2 is a potential strategy.

## 8. Prognosis

PMEC is a relatively inert tumor whose prognosis is usually considered optimistic. The 5-year OS of PMEC is approximately 45% to 70% in general [2, 8, 31, 73], although it is strongly influenced by the TMN stage and the pathological grade [2, 20, 27, 31]. The survival outcome of patients with PMEC seems to be better than that of patients with small-cell lung cancer (SCLC) and non-small-cell lung cancer (NSCLC) [20, 74, 75]. In the study by Cheng et al. [27], low-grade tumors are much more common in the younger age group, while high-grade ones are common in the older group. Huo et al. [8] concluded that age <50 years old, central/endobronchial growth pattern, tumor size <3 cm, low-grade tumor, Ki-67 labeling index <10%, and complete resection indicated a better OS and prognosis. Qiu et al. [20] drew a similar conclusion. They primarily employed CCS to predict survival curves, and their multivariate Cox analysis revealed that age >60 years, poor differentiation, tumor sizes >30 mm, lymph node metastases, and distant metastases were independent factors of a poor prognosis. In the study by Hsieh et al. [2], the tumor pathological grade influenced neither disease-free survival (DFS) nor OS, differing from previous studies. Park et al. [30] proposed this to be a factor of adverse prognosis. Tumors with greater than 6.5 SUV_max_ are more likely to be high-grade tumors, appeared more often in lymph nodes and distant metastasis, and deduced worse survival outcomes. Press et al. [41] showed that *HER2/neu* immunostaining and amplification were predictors of a poor prognosis independent of pathological grades, tumor sizes, and lymph node metastasis. Mukaigawa et al. [67] observed that the prognosis of PD-L1-positive patients was significantly worse. Moreover, PD-L1 expression is associated with poor DFS.

MECT1-MAML2 rearrangement is more common in low-grade PMEC. Similarly, the survival rate of patients with low-grade PMEC is significantly higher than that of patients with high-grade PMEC. Hence, MECT1-MAML2 rearrangement seems to indicate a better prognosis. However, some studies suggested that not only MECT1-MAML2 rearrangements were correlated with PMEC pathological grading, but also the translocation status was irrelevant to prognosis [8, 76].

In summary, age ≥ 50–60 years, tumor size ≥ 3 cm, poor pathological differentiation, Ki-67 labeling index ≥10%, SUV_max_ > 6.5, HER-2/neu immunostaining and amplification, PD-L1-positive, lymph node metastases, and distant metastases are associated with poor prognostic factors in PMEC.

## 9. Conclusion

PMEC, first reported by Smetana in 1952, is a rare primary pulmonary malignant neoplasm. Detailed results of clinical characteristics, epidemiological features, treatment, and prognosis are summarized in Supplementary Table 1. As the most common malignant salivary gland tumor, the survival outcomes of patients with PMEC seem to be better than those of patients with NSCLC and SCLC; however, accurate and early diagnosis plays critical roles. There is no clear diagnostic clinical symptom that has been related to PMEC. Most patients with PMEC present symptoms of bronchial obstruction, while several asymptomatic patients were only diagnosed with PMEC during a physical examination. CT is a necessary approach for the diagnosis and differential diagnosis: a tumor with well-defined mass and oval or round shape and a smooth margin, central type or hilar type, and marked enhancement more possibly diagnosed as PMEC. PMEC is histopathologically characterized by three cell types: squamous, intermediate, and mucus-secreting cells, classified into low-grade and high-grade histological appearance, mitotic frequency, cellular atypia, and necrocytosis. Immunohistochemical findings show that p63, p40, CK5/6, and CK7 are usually positive, while TTF-1 and napsin A are negative. A gene mutation in *t* (11; 19) (*q*21; *p*13) generates the fusion protein MECT1-MAML2, which is proposed to drive PMEC onset. Therefore, we can identify some diseases by using IHC and molecular examination. In the meantime, an EGFR mutation (exon 21 L861Q heterozygous mutation) is also certified to exist in PMEC. It has been demonstrated to be the molecular interpretation of EGFR signal activation that the MECT1-MAML2 fusion protein upregulates expression of AREG by direct binding to CREB. It provides clinical evidence for the effectiveness of TKI therapy. Some other gene mutations may lead to custom treatment options. It may provide new directions for future studies. There is temporarily no consolidated standard for treating PMEC, and surgical resection is the mainstay of treatment for low-grade PMEC. The effects of chemotherapy or radiotherapy are undefined. They could be used for patients with high-grade tumors with extrathoracic invasion. TKI therapy such as gefitinib and erlotinib had therapeutic responses. An immunotherapy approach for PMEC has powerful potential and needs to be explored in depth. The adverse prognostic factors are age ≥50–60, tumor size ≥ 3 cm, poor differentiation, Ki-67 labeling index ≥10%, SUV_max_ > 6.5, HER-2/neu immunostaining and amplification, PD-L1-positive, lymph node metastases, and distant metastases.

## Figures and Tables

**Table 1:** IHC results of the literature review.

Ref.	P63+	CK7+	Muc5Ac+	P40+	CK5/6+	TTF-1–	Napsin A–	HER2–	Ki-67
[8]	26/26	26/26	26/26	26/26	NM	26/26	NM	26/26	Median 4.1% (low-grade) Median 22.4% (high-grade)

[11]	25/25	NM	NM	23/25	NM	25/25	25/25	NM	NM

[17]	1/1	NM	NM	1/1	NM	NM	NM	NM	NM

[19]	5/5	6/6	NM	NM	2/5	6/6	2/2	NM	<10% (4/6 low-grade) ≥20% (2/6 high-grade)

[22]	1/1	NM	NM	1/1	NM	1/1	NM	NM	NM

[23]	NM	1/1	NM	1/1	1/1	1/1	1/1	NM	40% (high-grade)

Total.	58/58	33/33	26/26	52/54	3/6	59/59	28/28	26/26	
	100%	100%	100%	96.3%	50%	100%	100%	100%	

NM: No mention.

**Table 2:** The expression of PD-1, PD-L1, and PD-L2 in MEC.

Ref.	Number of cases	Positions	PD-1 (%)	PD-L1 (%)	PD-L2 (%)
[63]	41	Lungs	63.4	0	Low-grade: cytomembrane of squamous cells and intermediate cells shows medium or higher intensity (*N* = 8). High-grade: cytomembrane of squamous cells and intermediate cells shows focal positive (*N* = 33)
[64]	27	Salivary gland	81.5	25.9	63
[65]	9	Salivary gland	NM	55.6	NM
[66]	7	Salivary gland	NM	57.1	14.2
[67]	34	Salivary gland	NM	9	NM

## Supplementary Materials

Supplementary Materials Supplementary Table 1: detailed results of clinical characteristics, epidemiological features, diagnostic modality, the follow-up time, treatment, and prognosis of PMEC in the literature.

## References

[1] Smetana H. F., Iverson L., Swan L. L. (1952). Bronchogenic carcinoma; an analysis of 100 autopsy cases. *Mil Surg*.

[2] Hsieh C. C., Sun Y. H., Lin S. W., Yeh Y. C., Chan M. L. (2017). Surgical outcomes of pulmonary mucoepidermoid carcinoma: a review of 41 cases. *PLoS One*.

[3] Molina J. R., Aubry M. C., Lewis J. E. (2007). Primary salivary gland-type lung cancer: spectrum of clinical presentation, histopathologic and prognostic factors. *Cancer*.

[4] Song Z., Liu Z., Wang J., Zhu H., Zhang Y. (2013). Primary tracheobronchial mucoepidermoid carcinoma–a retrospective study of 32 patients. *World Journal of Surgical Oncology*.

[5] Wang Y., Cai S., Xue Q. (2020). Treatment outcomes of patients with tracheobronchial mucoepidermoid carcinoma compared with those with adenoid cystic carcinoma. *European Journal of Surgical Oncology*.

[6] Leonardi H. K., Jung-Legg Y., Legg M. A., Neptune W. B. (1978). Tracheobronchial mucoepidermoid carcinoma. Clinicopathological features and results of treatment. *The Journal of Thoracic and Cardiovascular Surgery*.

[7] Roden A. C. (2021). Recent updates in salivary gland tumors of the lung. *Seminars in Diagnostic Pathology*.

[8] Huo Z., Wu H., Li J. (2015). Primary pulmonary mucoepidermoid carcinoma: histopathological and moleculargenetic studies of 26 cases. *PLoS One*.

[9] Yousem S. A., Hochholzer L. (1987). Mucoepidermoid tumors of the lung. *Cancer*.

[10] Salem A., Bell D., Sepesi B. (2017). Clinicopathologic and genetic features of primary bronchopulmonary mucoepidermoid carcinoma: the MD Anderson Cancer Center experience and comprehensive review of the literature. *Virchows Archiv*.

[11] Roden A. C., García J. J., Wehrs R. N. (2014). Histopathologic, immunophenotypic and cytogenetic features of pulmonary mucoepidermoid carcinoma. *Modern Pathology*.

[12] Yu Y., Song Z., Gao H. (2012). EGFR L861Q mutation is a frequent feature of pulmonary mucoepidermoid carcinoma. *Journal of Cancer Research and Clinical Oncology*.

[13] Xi J. J., Jiang W., Lu S. H., Zhang Cy, Fan H., Wang Q. (2012). Primary pulmonary mucoepidermoid carcinoma: an analysis of 21 cases. *World Journal of Surgical Oncology*.

[14] Lee K. W., Chan A. B., Lo A. W., Lam K. C. (2011). Erlotinib in metastatic bronchopulmonary mucoepidermoid carcinoma. *Journal of Thoracic Oncology*.

[15] Han S. W., Kim H. P., Jeon Y. K. (2008). Mucoepidermoid carcinoma of lung: potential target of EGFR-directed treatment. *Lung Cancer*.

[16] Rossi G., Sartori G., Cavazza A., Tamberi S. (2009). Mucoepidermoid carcinoma of the lung, response to EGFR inhibitors, EGFR and K-RAS mutations, and differential diagnosis. *Lung Cancer*.

[17] Abdalla M., Sinyagovskiy P., Mohamed W., Abdelghani A., Al-azzam B. (2018). A rare case of pulmonary mucoepidermoid carcinoma in an 81-year-old male. *Am J Case Rep*.

[18] Chin C. H., Huang C. C., Lin M. C., Chao T. Y., Liu S. F. (2008). Prognostic factors of tracheobronchial mucoepidermoid carcinoma–15 years experience. *Respirology*.

[19] Hou J., Wang H., Zhang G., Huang Y., Ma Z. (2017). Mucoepidermoid carcinoma of the lung: report of 29 cases. *Zhongguo Fei Ai Za Zhi*.

[20] Qiu L., Song P., Chen P. (2021). Clinical characteristics and prognosis of patients with pulmonary mucoepidermoid carcinoma: a SEER-based analysis. *Frontiers in Oncology*.

[21] Shen C., Che G. (2014). Clinicopathological analysis of pulmonary mucoepidermoid carcinoma. *World Journal of Surgical Oncology*.

[22] Omesh T., Gupta R., Saqi A., Burack J., Khaja M. (2018). A rare case of endobronchial mucoepidermoid carcinoma of the lung presenting as non-resolving pneumonia. *Respiratory Medicine Case Reports*.

[23] Yan H., Li X., Peng Y., Zhang P., Zou N., Liu X. (2020). Apatinib and fractionated stereotactic radiotherapy for the treatment of limited brain metastases from primary lung mucoepidermoid carcinoma: a case report. *Medicine (Baltimore)*.

[24] Kalhor N., Moran C. A. (2018). Pulmonary mucoepidermoid carcinoma: diagnosis and treatment. *Expert Review of Respiratory Medicine*.

[25] Kumar V., Soni P., Garg M. (2018). A comparative study of primary adenoid cystic and mucoepidermoid carcinoma of lung. *Frontiers in Oncology*.

[26] Ban X., Shen X., Hu H. (2021). Predictive CT features for the diagnosis of primary pulmonary mucoepidermoid carcinoma: comparison with squamous cell carcinomas and adenocarcinomas. *Cancer Imaging*.

[27] Cheng D. L., Hu Y. X., Hu P. Q., Wen G., Liu K. (2017). Clinicopathological and multisection CT features of primary pulmonary mucoepidermoid carcinoma. *Clinical Radiology*.

[28] Han X., Zhang J., Fan J., Cao Y., Gu J., Shi H. (2019). Radiological and clinical features and outcomes of patients with primary pulmonary salivary gland-type tumors. *Canadian Respiratory Journal*.

[29] Wang Y. Q., Mo Y. X., Li S., Luo R. Z., Mao S. Y., Shen J. X. (2015). Low-grade and high-grade mucoepidermoid carcinoma of the lung: CT findings and clinical features of 17 cases. *American Journal of Roentgenology*.

[30] Park B., Kim H. K., Choi Y. S. (2015). Prediction of pathologic grade and prognosis in mucoepidermoid carcinoma of the lung using ^1^⁸F-fdg PET/CT. *Korean Journal of Radiology*.

[31] Zhang X. P., Hu P. Z., Shen S. S., Li X. Y. (2018). Clinical characteristics and prognostic analyses of 87 patients with pulmonary mucoepidermoid carcinoma. *Zhonghua Zhongliu Zazhi*.

[32] Ishizumi T., Tateishi U., Watanabe S. I., Matsuno Y. (2008). Mucoepidermoid carcinoma of the lung: high-resolution CT and histopathologic findings in five cases. *Lung Cancer*.

[33] Vadasz P., Egervary M. (2000). Mucoepidermoid bronchial tumors: a review of 34 operated cases. *European Journal of Cardio-Thoracic Surgery*.

[34] Brandwein M. S., Ivanov K., Wallace D. I. (2001). Mucoepidermoid carcinoma: a clinicopathologic study of 80 patients with special reference to histological grading. *The American Journal of Surgical Pathology*.

[35] Kang D., Kim H., Jang S. (2013). Surgical outcomes of pulmonary mucoepidermoid carcinoma: a review of 23 cases. *The Thoracic and Cardiovascular Surgeon*.

[36] Zhu F., Liu Z., Hou Y. (2013). Primary salivary gland-type lung cancer: clinicopathological analysis of 88 cases from China. *Journal of Thoracic Oncology*.

[37] Wang M., Ouyang S., Sun P., Li D., Huang G. (2015). Pulmonary mucoepidermoid carcinoma in Chinese population: a clinicopathological and radiological analysis. *International Journal of Clinical and Experimental Pathology*.

[38] Kim N. I., Lee J. S. (2020). Greater specificity of p40 compared with p63 in distinguishing squamous cell carcinoma from adenocarcinoma in effusion cellblocks. *CytoJournal*.

[39] Sams R. N., Gnepp D. R. (2013). P63 expression can be used in differential diagnosis of salivary gland acinic cell and mucoepidermoid carcinomas. *Head and Neck Pathology*.

[40] Clauditz T. S., Reiff M., Gravert L. (2011). Human epidermal growth factor receptor 2 (HER2) in salivary gland carcinomas. *Pathology*.

[41] Press M. F., Pike M. C., Hung G. (1994). Amplification and overexpression of HER-2/neu in carcinomas of the salivary gland: correlation with poor prognosis. *Cancer Research*.

[42] Nakano T., Yamamoto H., Hashimoto K. (2013). HER2 and EGFR gene copy number alterations are predominant in high-grade salivary mucoepidermoid carcinoma irrespective of MAML2 fusion status. *Histopathology*.

[43] Shang J., Shui Y., Sheng L., Wang K., Hu Q., Wei Q. (2008). Epidermal growth factor receptor and human epidermal growth receptor 2 expression in parotid mucoepidermoid carcinoma: possible implications for targeted therapy. *Oncology Reports*.

[44] Haddad R., Colevas A. D., Krane J. F. (2003). Herceptin in patients with advanced or metastatic salivary gland carcinomas. A phase II study. *Oral Oncology*.

[45] Achcar R. D. O. D., Nikiforova M. N., Dacic S., Nicholson A. G., Yousem S. A. (2009). Mammalian mastermind like 2 11q21 gene rearrangement in bronchopulmonary mucoepidermoid carcinoma. *Human Pathology*.

[46] Wu L., Sun T., Kobayashi K., Gao P., Griffin J. D. (2002). Identification of a family of mastermind-like transcriptional coactivators for mammalian notch receptors. *Molecular and Cellular Biology*.

[47] Tonon G., Modi S., Wu L. (2003). t(11;19)(q21;p13) translocation in mucoepidermoid carcinoma creates a novel fusion product that disrupts a Notch signaling pathway. *Nature Genetics*.

[48] Wu L., Liu J., Gao P. (2005). Transforming activity of MECT1-MAML2 fusion oncoprotein is mediated by constitutive CREB activation. *EMBO Journal*.

[49] Wang K., McDermott J. D., Schrock A. B. (2017). Comprehensive genomic profiling of salivary mucoepidermoid carcinomas reveals frequent BAP1, PIK3CA, and other actionable genomic alterations. *Annals of Oncology*.

[50] Macarenco R. S., Uphoff T. S., Gilmer H. F. (2008). Salivary gland-type lung carcinomas: an EGFR immunohistochemical, molecular genetic, and mutational analysis study. *Modern Pathology*.

[51] Yamamoto T., Nakajima T., Suzuki H. (2016). Surgical treatment of mucoepidermoid carcinoma of the lung: 20 years’ experience. *Asian Cardiovascular & Thoracic Annals*.

[52] Chenevert J., Barnes L. E., Chiosea S. I. (2011). Mucoepidermoid carcinoma: a five-decade journey. *Virchows Archiv*.

[53] Qin B. D., Jiao X. D., Liu K. (2018). Clinical, pathological and treatment factors associated with the survival of patients with primary pulmonary salivary gland-type tumors. *Lung Cancer*.

[54] Li X., Yi W., Zeng Q. (2018). CT features and differential diagnosis of primary pulmonary mucoepidermoid carcinoma and pulmonary adenoid cystic carcinoma. *Journal of Thoracic Disease*.

[55] Falk N., Weissferdt A., Kalhor N., Moran C. A. (2016). Primary pulmonary salivary gland-type tumors: a review and update. *Advances in Anatomic Pathology*.

[56] García J. J., Jin L., Jackson S. B. (2015). Primary pulmonary hyalinizing clear cell carcinoma of bronchial submucosal gland origin. *Human Pathology*.

[57] Takamatsu M., Sato Y., Muto M. (2018). Hyalinizing clear cell carcinoma of the bronchial glands: presentation of three cases and pathological comparisons with salivary gland counterparts and bronchial mucoepidermoid carcinomas. *Modern Pathology*.

[58] Chapman E., Skalova A., Ptakova N. (2018). Molecular profiling of hyalinizing clear cell carcinomas revealed a subset of tumors harboring a novel EWSR1-CREM fusion: report of 3 cases. *The American Journal of Surgical Pathology*.

[59] Sonobe S., Inoue K., Tachibana S. (2014). A case of pulmonary mucoepidermoid carcinoma responding to carboplatin and paclitaxel. *Japanese Journal of Clinical Oncology*.

[60] O’Neill I. D. (2009). Gefitinib as targeted therapy for mucoepidermoid carcinoma of the lung: possible significance of CRTC1-MAML2 oncogene. *Lung Cancer*.

[61] Chen Z., Chen J., Gu Y. (2014). Aberrantly activated AREG-EGFR signaling is required for the growth and survival of CRTC1-MAML2 fusion-positive mucoepidermoid carcinoma cells. *Oncogene*.

[62] Kim Y., Lee S. J., Lee J. Y. (2017). Clinical trial of nintedanib in patients with recurrent or metastatic salivary gland cancer of the head and neck: a multicenter phase 2 study (Korean Cancer Study Group HN14-01). *Cancer*.

[63] Liu J., Wu L., Lin Q. (2018). Expression of programmed death receptor 1 amd its ligand in primary lung mucoepidermoid carcinoma and its clinical significance. *Fujian Med J*.

[64] Chang H., Kim J. S., Choi Y. J. (2017). Overexpression of PD-L2 is associated with shorter relapse-free survival in patients with malignant salivary gland tumors. *OncoTargets and Therapy*.

[65] Harada K., Ferdous T., Ueyama Y. (2018). PD-L1 expression in malignant salivary gland tumors. *BMC Cancer*.

[66] Nakano T., Takizawa K., Uezato A., Taguchi K., Toh S., Masuda M. (2019). Prognostic value of programed death ligand-1 and ligand-2 co-expression in salivary gland carcinomas. *Oral Oncology*.

[67] Mukaigawa T., Hayashi R., Hashimoto K., Ugumori T., Hato N., Fujii S. (2016). Programmed death ligand-1 expression is associated with poor disease free survival in salivary gland carcinomas. *Journal of Surgical Oncology*.

[68] Cohen R. B., Delord J. P., Doi T. (2018). Pembrolizumab for the treatment of advanced salivary gland carcinoma: findings of the phase 1b KEYNOTE-028 study. *American Journal of Clinical Oncology*.

[69] Chan T. A., Yarchoan M., Jaffee E. (2019). Development of tumor mutation burden as an immunotherapy biomarker: utility for the oncology clinic. *Annals of Oncology*.

[70] Cavalieri S., Platini F., Bergamini C. (2019). Genomics in non-adenoid cystic group of salivary gland cancers: one or more druggable entities?. *Expert Opinion on Investigational Drugs*.

[71] Kurzrock R., Bowles D. W., Kang H. (2020). Targeted therapy for advanced salivary gland carcinoma based on molecular profiling: results from MyPathway, a phase IIa multiple basket study. *Annals of Oncology*.

[72] Theocharis S., Tasoulas J., Masaoutis C., Kokkali S., Klijanienko J. (2020). Salivary gland cancer in the era of immunotherapy: can we exploit tumor microenvironment?. *Expert Opinion on Therapeutic Targets*.

[73] Lassche G., van Boxtel W., Ligtenberg M. J. L., van Engen-van Grunsven A. C., van Herpen C. M. (2019). Advances and challenges in precision medicine in salivary gland cancer. *Cancer Treatment Reviews*.

[74] Saglietti C., Volante M., La Rosa S. (2017). Cytology of primary salivary gland-type tumors of the lower respiratory tract: report of 15 cases and review of the literature. *Frontiers of Medicine*.

[75] Fischer B., Arcaro A. (2008). Current status of clinical trials for small cell lung cancer. *Reviews on Recent Clinical Trials*.

[76] Saade R. E., Bell D., Garcia J., Roberts D., Weber R. (2016). Role of CRTC1/MAML2 translocation in the prognosis and clinical outcomes of mucoepidermoid carcinoma. *JAMA Otolaryngol Head Neck Surg*.

