# Supplementary material for: Pulmonary Salivary Gland Tumor, Mucoepidermoid Carcinoma: A Literature Review
Source: J Oncol. 2022 Nov 2;2022:9742091. doi: 10.1155/2022/9742091 (PMC9646301; doi:10.1155/2022/9742091)
Supplement: Supplementary Materials — Supplementary Table 1: detailed results of clinical characteristics, epidemiological features, diagnostic modality, the follow-up time, treatment, and prognosis of PMEC in the literature. [file 9742091.f1.pdf]

Supplementary Table 1. Clinical data of PMEC reported in the literatures.

Ref.	Period (years)	Number of cases	Mean age (years)	Mean size (cm)	Gender		History of smoking		Diagnostic modality	Grade		Treatment	The median follow-up time (months)	5-y OS (%)
					M	F	Y	N		low	high			
[2]	1991-2015	41	61.4	3	30	11	21	20	Resection 41	10	31	Surgery 41	42.9	57.9
[8]	2000-2014	26	46.5	2.5	13	13	7	19	Resection 23<br>Lung biopsy 3	18	8	Surgery 23<br>RT and/or CHT 23	NM	72.1
[10]	NM	16	40.4	2.6	7	9	8	8	NM	14	2	Surgery 14<br>RT or CHT 9	40.8	NM
[13]	2004-2011	21	43.4	NM	10	11	2	21	Resection 21	17	4	Surgery 21<br>Adjuvant CHT or RT 2	Low grade 46.6<br>High grade 41.5	NM
[19]	2006-2015	29	45	4.8	18	11	8	21	Resection 16<br>Lung biopsy 6<br>Lymph node biopsy 2<br>bronchoscopic biopsy 5	12	17	Surgery 17<br>Adjuvant therapy 16	35	39.4
[29]	2005-2013	17	44.35 ± 17.10	3.1	10	7	NM	NM	Resection 16<br>bronchoscopic biopsy 1	11	6	NM	30.6	NM
[51]	1993-2012	9	54	NM	2	7	NM	NM	Resection 9	6	3	Surgery 9	39.8	72.9
Total.		159			90	69	46	89		88	71			
%					56.6	43.3	34.1	65.9		55.3	44.7			

M: Male, F: Female, Y: Yes, N: No, RT: Radiotherapy, CHT: Chemotherapy
